# Bioinformatics methods in biomarkers of preeclampsia and associated potential drug applications

**DOI:** 10.1186/s12864-022-08937-3

**Published:** 2022-10-19

**Authors:** Ying Peng, Hui Hong, Na Gao, An Wan, Yuyan Ma

**Affiliations:** 1 Department of Obstetrics and Gynecology, Qilu Hospital of Shandong University, Jinan, Shandong China; 2 Department of Obstetrics and Gynecology, The First Affiliated Hospital of USTC, Division of Life Sciences and Medicine，University of Science and Technology of China, Hefei, Anhui China; 3 Department of Obstetrics and Gynecology, Qilu Hospital of Shandong University, Jinan Shandong, 250012 China

**Keywords:** Preeclampsia, Bioinformatics analysis, Differentially expressed genes, Immune mechanism, Molecular markers, Potential drugs

## Abstract

**Background:**

Preeclampsia is a pregnancy-related condition that causes high blood pressure and proteinuria after 20 weeks of pregnancy. It is linked to increased maternal mortality, organ malfunction, and foetal development limitation. In this view, there is a need critical to identify biomarkers for the early detection of preeclampsia. The objective of this study is to discover critical genes and explore medications for preeclampsia treatment that may influence these genes.

**Methods:**

Four datasets, including GSE10588, GSE25906, GSE48424 and GSE60438 were retrieved from the Gene Expression Omnibus database. The GSE10588, GSE25906, and GSE48424 datasets were then removed the batch effect using the “sva” R package and merged into a complete dataset. The differentially expressed genes (DEGs) were identified using the “limma” R package. The potential small-molecule agents for the treatment of PE was further screened using the Connective Map (CMAP) drug database based on the DEGs. Further, Weight gene Co-expression network (WGNCA) analysis was performed to identified gene module associated with preeclampsia, hub genes were then identified using the logistic regression analysis. Finally, the immune cell infiltration level of genes was evaluated through the single sample gene set enrichment analysis (ssGSEA).

**Results:**

A total of 681 DEGs (376 down-regulated and 305 up-regulated genes) were identified between normal and preeclampsia samples. Then, Dexamethasone, Prednisone, Rimexolone, Piretanide, Trazodone, Buflomedil, Scoulerin, Irinotecan, and Camptothecin drugs were screened based on these DEGs through the CMAP database. Two modules including yellow and brown modules were the most associated with disease through the WGCNA analysis. KEGG analysis revealed that the chemokine signaling pathway, Th1 and Th2 cell differentiation, B cell receptor signalling pathway and oxytocin signalling pathway were significantly enriched in these modules. Moreover, two key genes, PLEK and LEP were evaluated using the univariate and multivariate logistic regression analysis from the hub modules. These two genes were further validated in the external validation cohort GSE60438 and qRT-PCR experiment. Finally, we evaluated the relationship between immune cell and two genes.

**Conclusion:**

In conclusion, the present study investigated key genes associated with PE pathogenesis that may contribute to identifying potential biomarkers, therapeutic agents and developing personalized treatment for PE.

**Supplementary Information:**

The online version contains supplementary material available at 10.1186/s12864-022-08937-3.

## Introduction

Preeclampsia (PE) is a pregnancy disorder that causes high blood pressure and proteinuria [1] after 20 weeks of pregnancy [2].It affects 3%-10% of all pregnancies [3–5], and is associated with substantial maternal morbidity, mortality, organ dysfunction, iatrogenic premature delivery [6], and foetal development restriction [7]. Preeclampsia affects the brain development and function of the offspring, increasing the risk of intellectual disability [8], epilepsy [9], autism [10], and schizophrenia [11, 12]. Women with a history of PE are more likely to develop cardiovascular disease or hypertension,^5^ resulting in financial and psychological consequences on the family and society. There is no obvious therapeutic intervention for PE today; the only effective treatment is pregnancy, which might result in low birth weight and long-term detrimental health implications for infants [13, 14]. Early assessment of PE risk is critical for high-risk pregnant women because it allows for the implementation of preventive strategies to lower the incidence of PE and improve maternal and infant outcomes.

However, the etiology and pathogenesis of PE are yet unknown. Several hypotheses such as maternal-foetal (paternal) immune maladjustment [15] and inflammatory cytokine disorders [16], all suggest that PE is associated with a number of risk factors, including obesity, hypertension, diabetes, oxidative stress, foetal rejection, genetic polymorphism inheritance [17], and trophoblast insufficiency [18, 19]. The main causes are identified as immunological intolerance [20, 21] and angiogenesis imbalance, and numerous research has demonstrated the immune mechanism of PE development. The latter causes PE by generating an imbalance in immune tolerance at the mother-infant interaction [22, 23]. The pathophysiological basis of PE is shallow placental implantation. The mutual adaptation of villous trophoblast cells and the mother's immune system is required for effective placental development. The poor placental formation will result in acute-like graft rejection disease under the influence of specific immunological factors generating immune intolerance in the mother and child.

Immune system modifications have been widely recognized as the key determinants of PE [24], which is a systemic inflammatory response resulting in an imbalance between placental substances and the corresponding adaptation of the mother[25, 26]. Shah et al. [27] in their study compared the expression of CD66B, nuclear factor NF-κB, and cyclooxygenase-2 (COX-2) in the subcutaneous fat of women with PE (*n* = 7), normal pregnant women (*n* = 6), and normal non-pregnant women (*n* = 5). The percentages of CD66B, NF-κB, and COX-2 in PE patients were significantly higher than in normal non-pregnant or normal pregnant patients. Moreover, Xu et al. [28]. discovered five hub genes that are associated with immune cells in the immunologic microenvironment at the maternal-foetal interface.

An increasing number of researchers are actively pursuing molecular markers using data mining and analysis of databases to the diagnosis and treatment of PE. However, most recent studies using single dataset to identify the key genes and lack of external validation cohort, which may induce a unstable and unreliable result. For example, Liu et al. using the GSE60438 dataset to identify 17 hub genes through the differentially expressed analysis, protein–protein network and svm analysis, while lack of external cohort [29]. Lin et al. using the GSE48424 dataset to identify three key genes (HDC, MS4A2 and SLC18A2) associated with PE through PPI network analysis. Although they applied GSE149437 to verify the predictive value of the above these genes, the result still lack reliability [30]. Moreover, Wang et al. explored the immune cell infiltration in PE, however, the dataset that used in the study is relatively small, and lack of the stability [31].

In this study, we aimed to investigate the potential biomarkers, immune cell infiltration and drug in PE via integrative multiple datasets. These results were further validated using the qRT-PCR experiment. Our findings might provide the novel biomarkers for the prediction and diagnosis of PE.

## Methods

### Selection of the GEO dataset and data processing

**Fig. 1:**
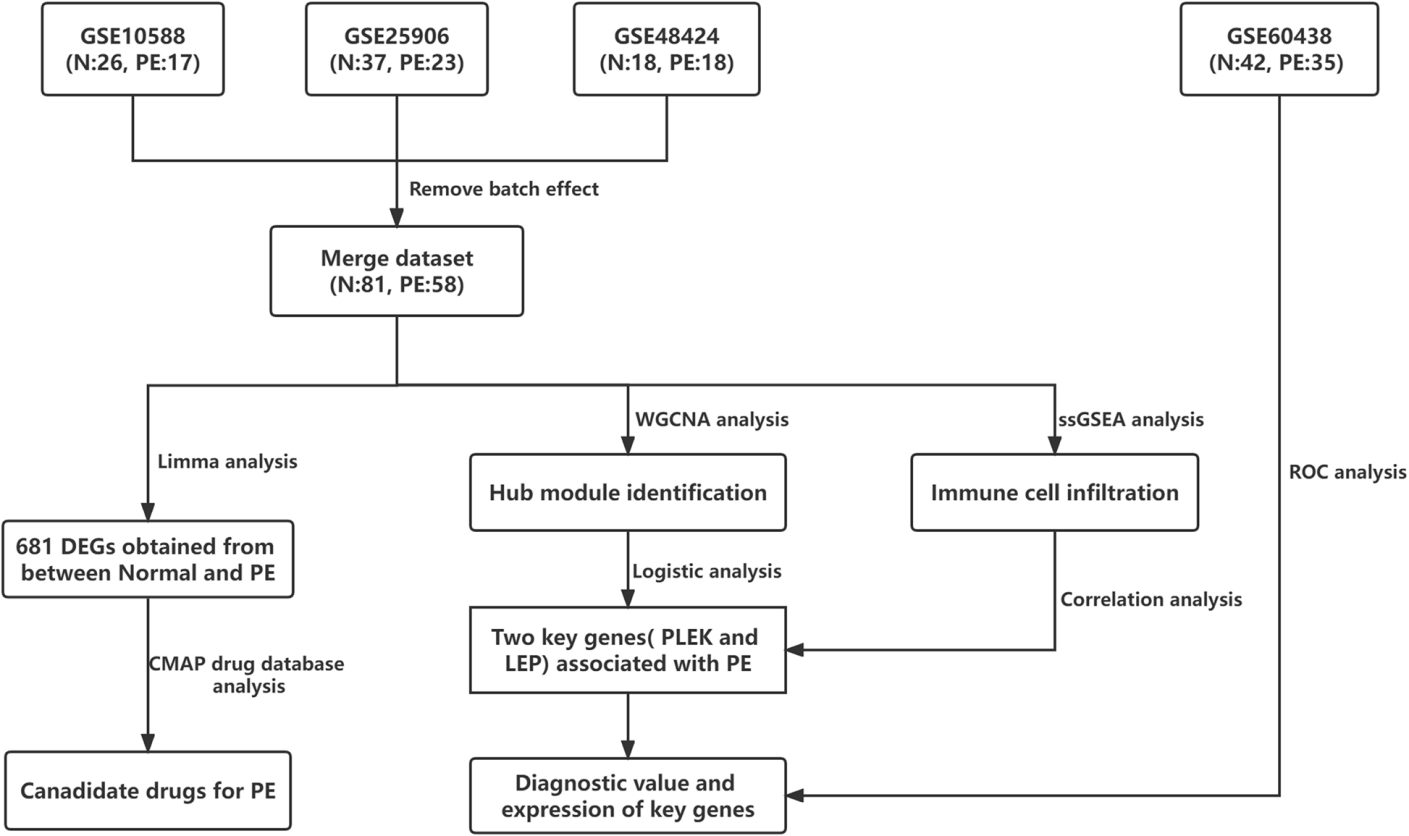
The overall design of the study

The gene expression microarray datasets GSE10588, GSE25906, GSE48424, and GSE60438 were downloaded from the GEO database (http://www.ncbi.nlm.nih.gov/geo/). GSE10588 contains 26 normal placental tissues and 17 preeclampsia placental tissues [32], GSE25906 contains 37 normal placental tissues and 23 preeclampsia placental tissues [33], GSE48424 contains 18 normal blood samples and 18 preeclampsia blood samples [34], and GSE60438 contains 42 normal samples and 35 PE samples [35]. To verify data reliability, we used the combat function in the ‘SVA’ R package to eliminate the batch effect from three datasets (GSE10588, GSE25906, and GSE48424) and combined these three datasets (Merge dataset). The distribution of datasets before and after the merger was then evaluated using principal component analysis. The dataset GSE60438 was selected for external validation [35]. The detail pipeline of this study was showed in Fig. 1.

### Differential expression analysis

We used the ‘Limma’ R package (version 4.0; http://www.bioconductor.org/packages/release/bioc/html/limma.html) to discover differential genes between normal and PE samples in a combined dataset of 81 normal samples and 58 PE samples. For the screening criterion, corrected *P*-values less than 0.05 and log_2_ |Fold Change|> 0 were considered statistically significant.

### CMAP drug database analysis

To find potential small-molecule agents for the treatment of PE, the top 1000 up-regulated and down-regulated DEGs were uploaded to the Connective Map database (CMAP) (http://www.broadinstitute.org/cmap/).

### Co-expression network analysis of weighted genes

The “WGCNA” R package was used for co-expression analysis. The genes with the largest variance (25%) were firstly selected to assure the heterogeneity and then filtered to remove outliers using sample clustering methods. The co-expression similarity matrix included absolute values of correlations between transcription expression levels. A Pearson correlation matrix was constructed for matching genes. We used the power function amn =| key | beta (key = the Pearson correlation between gene m and n; AMN = adjacency between gene M and gene N) to construct a weighted adjacency matrix. The parameter β highlighted genes with significant correlations while penalizing those with weak correlations. Following that, the appropriate β value was selected to perform the similarity matrix and investigate the scale-free co-expression network. The adjacency matrix was then transformed into a topological overlap matrix (TOM), which calculated the network connectedness of the genes in the topology matrix as the sum of the adjacent genes generated by all the other networks. Based on TOM similarity data, average linkage level clustering was performed, and the minimum size of the gene tree (genome) was selected 30. The gene significance (GS) was calculated to quantify the correlation between genes and sample traits to determine the value of each module. Module eigengenes (MEs) were regarded as the major components in the principal component analysis of each gene module, and the expression patterns of all genes could be summed as a single typical expression profile within a specific module. The log_10_ conversion of p-value in linear regression between gene expression and clinical data (GS = lgP) was designated as GS. Modular significance (MS) was defined as the average GS within a module, reflecting the correlation between the module and the sample features. We established a threshold value (< 0.25) to increase the capacity of the modules by merging several modules of comparable height. Normal and PE samples were selected as clinical phenotypes. The gene modules were then examined alongside the clinical phenotypes. After identifying the relevant modules, we calculated GS and MM values (module membership [MM] is the correlation between the genes of the module and gene expression profile) and set the threshold values for each key gene.

### GO and KEGG enrichment analysis

The R package of ‘clusterProfiler’ to perform Gene ontology (GO) and the Kyoto Encyclopedia of Genes and Genomes (KEGG) [36–38]enrichment analysis to uncover the function of genes and potential pathways in the module. Furthermore, the screening criteria for significant functions and pathways were adjusted p-value less than 0.05.

### Logistic regression

To identified key genes associated with PE, we firstly constructed PPI network based on the hub modules. We then using the MCODE algorithm and logistic regression analysis to screened key genes from the PPI network. The receiver operating characteristic (ROC) curve analysis was applied to the accuracy of the diagnostic model from the result of logistic regression analysis in the merge dataset and external validation dataset.

### Immune infiltration analysis

The immune cell gene sets were retrieved from previous related literature, and then used to assess the immune cell infiltration between normal and PE samples [28]. The function of immune-related gene sets was acquired to enrich gene sets and calculate the concentration of each sample score, standardized between 0 and 1, using the single-sample Gene Set Enrichment Analysis (ssGSEA) algorithm [39].

### Collection of human tissue specimens

A total of 60 placenta specimens were collected between August 2021 and January 2022, 30 specimens from patients with PE and 30 from healthy pregnant women at the The First Affiliated Hospital of USTC, Division of Life Sciences and Medicine, University of Science and Technology of China (Anhui Provincial Hospital) (Hefei, China). Written informed consent was obtained from all participants. This study was approved by The First Affiliated Hospital of USTC, Division of Life Sciences and Medicine, University of Science and Technology of China (Anhui Provincial Hospital) research ethics board (NO:2021KY161) and is based on the ethical requirements of the Helsinki Declaration. All participants have the right to know.

### Detection of the mRNA expression of the hub genes by RT-PCR

Tissue RNA was extracted using TRIzol® (Invitrogen; Thermo Fisher Scientific, Inc.) to assess the expression of important genes in the placentas of PE patients and healthy pregnant women. Total RNA was reverse transcribed into cDNA at 42˚C for 30 min using PrimeScript™ RT Master Mix (Takara Biotechnology Co., Ltd.). Subsequently, qPCR was performed on a CFX96™ Real-Time PCR Detection system (Bio-Rad Laboratories, Inc.) using TB Green™ Fast qPCR mix. The 2-ΔΔCq method was used to quantify the relative mRNA levels for the gene expression analysis [40]. The values were adjusted to endogenous GAPDH expression, and each experiment was repeated three times separately. Critical Technologies: GAPDH sense, 5′-GAAGGTGAAGGTCGGAGTCAA-3′; antisense 5′-CTGGAAGATGGTGATGGGATTT-3′; PLEK sense, 5′-AGATGCCTGGGTTCGGGATA-3′; antisense, 5′-GGTTTCTGGCAGTCGAATGGA-3′; LEP sense, 5′-GCTGTGCCCATCCAAAAAGT-3′; antisense, 5′-CCAGGAATGAAGTCCAAACCG-3′. The thermocycling conditions were as follows: 95˚C for 30 s, followed by 40 cycles of 95˚C for 5 s and 60˚C for 30 s.

## Statistical analysis

All RT-PCR experimental data were analyzed using GraphPad Prism 7.0. The expression level of each hub gene was expressed as a fold change using the 2^−ΔΔC^ method. *P* < 0.05 was considered significant.

## Results

### Identification of differential genes in PE

**Fig. 2:**
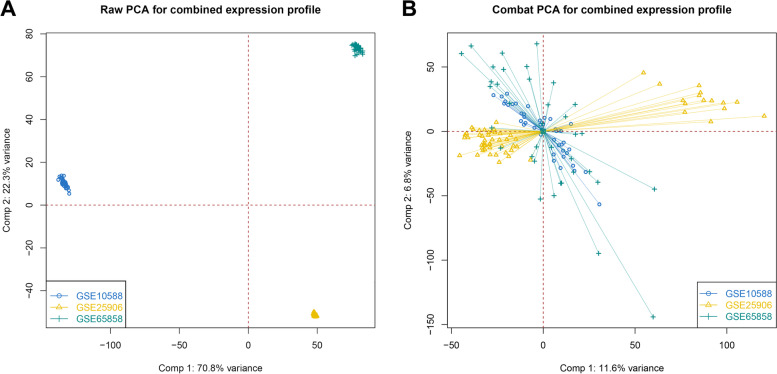
Evaluation of the batch effect before (**A**) and after (**B**) merging through the principal component analysis

**Fig. 3:**
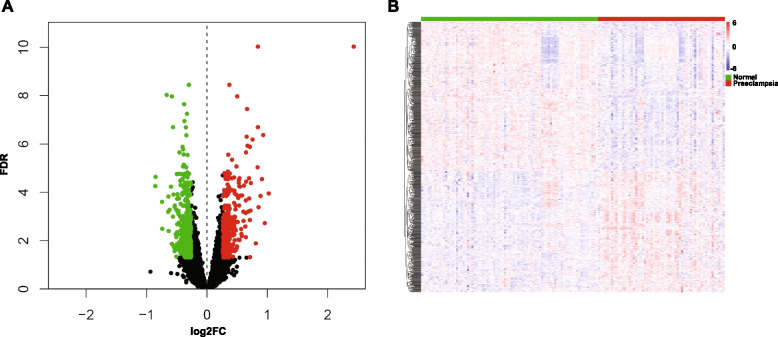
Identification of differentially expressed genes in the merged dataset. **A** Volcano plot of the genes, the green dots represent the down-regulated genes, red dots represent the up-regulated genes, while the black dots showed genes with no significance. **B** A heatmap plot of the differentially expressed genes

GSE10588, GSE25906, and GSE48424 datasets were pooled. We obtained a dataset with 81 normal samples and 58 PE samples after removing the batch effect. As shown in Figs. 2A and B, the datasets were discrete before merging. while the batch effect was removed, we discovered that the datasets were merged, showing that the batch effect was successfully erased. By comparing normal and PE samples, we found 376 down-regulated genes and 305 up-regulated genes (Figs. 3A and B). Figure 3A depicts up-down-regulated differential genes; down-regulated genes are denoted by green and the up-regulated genes are denoted by red point. Moreover, a heat map of the differentially regulated genes is shown in Fig. 3B.

### Identification of potential drugs for PE

**Fig. 4:**
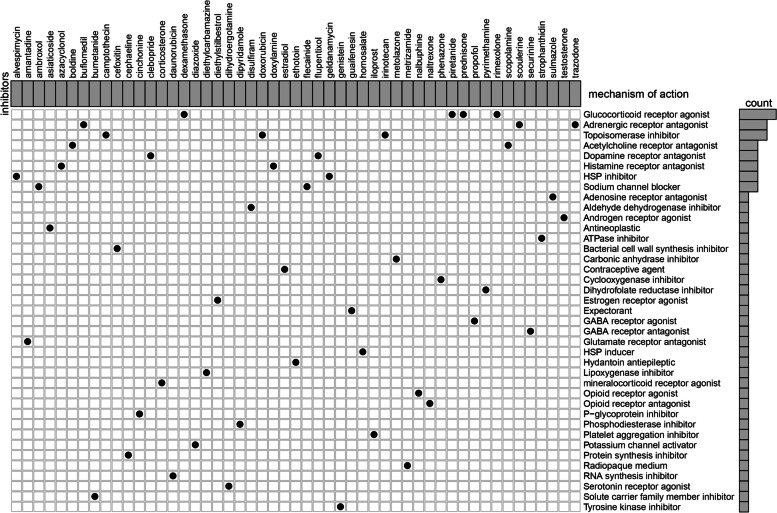
Identification of the molecular compounds with their mode of action using the CMAP database

Based on these DEGs, we looked into potential drug targeting pathways in the CMAP database. The analyses of the mechanism of action in the CMAP database revealed 39 mechanisms of action of 51 drugs. As illustrated in Fig. 4, dexamethasone, prednisone, rimexolone, and piretanide interacted via the glucocorticoid receptor agonist, whereas Trazodone, buflomedil, and scoulerine interacted via the adrenergic receptor antagonist. The co-action mechanism of irinotecan, camptothecin, and doxorubicin, on the other hand, was via topoisomerase inhibitor. These results may provide new insights for the treatment of PE.

### Modular identification and key gene screening for PE

**Fig. 5:**
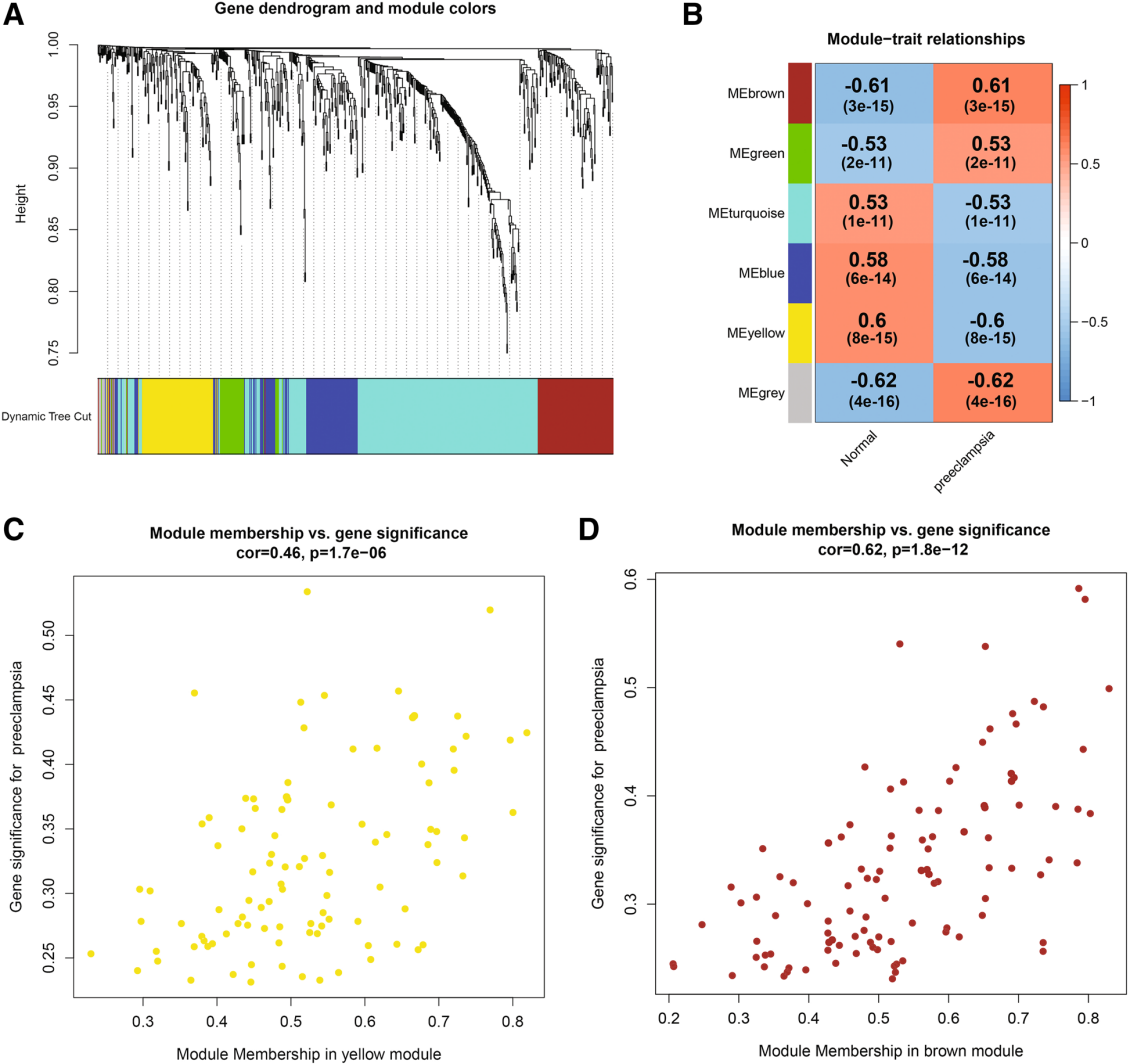
Identification of hub modules using the WGCNA analysis. **A** The dendrogram of the co-expression network was clustered based on the dissimilarity. **B** The module-trait heatmap showing the correlation between module eigengenes (ME) and traits. **C** Scatter plot of the red module. **D** Scatter plot of the brown module

**Fig. 6:**
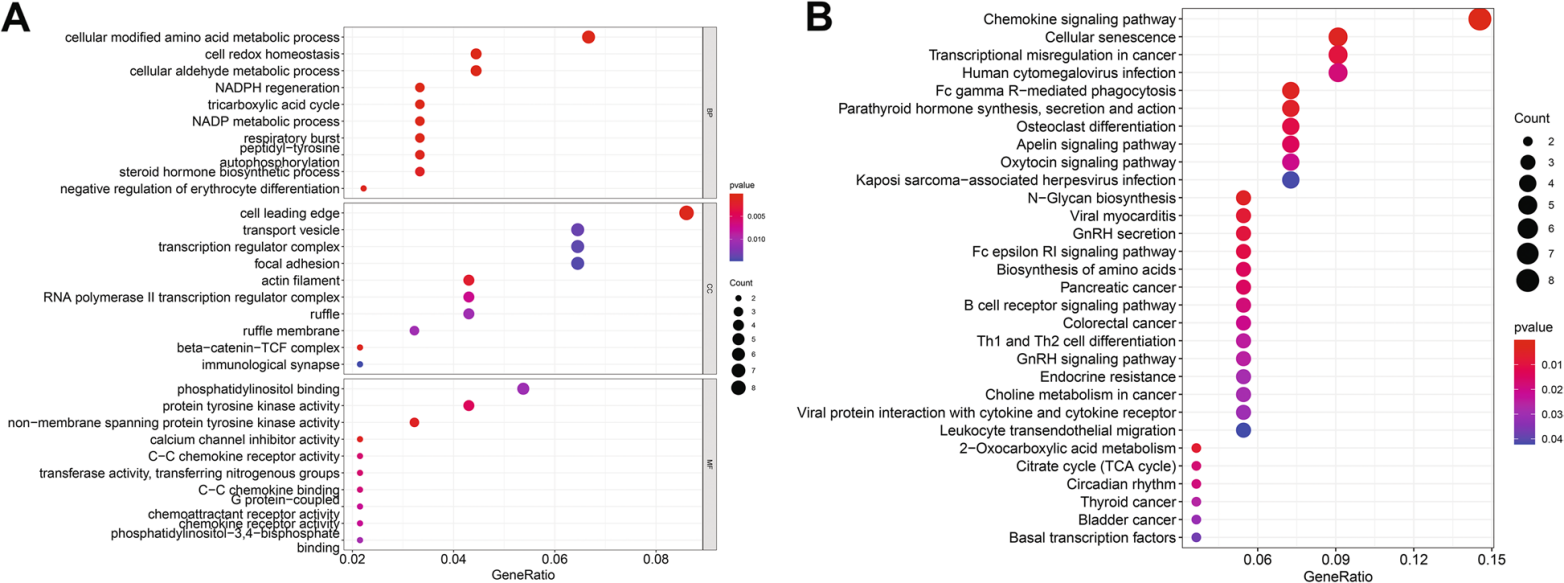
Gene ontology enrichment (**A**) and KEGG (**B**) enrichment analysis were performed in the yellow modules

**Fig. 7:**
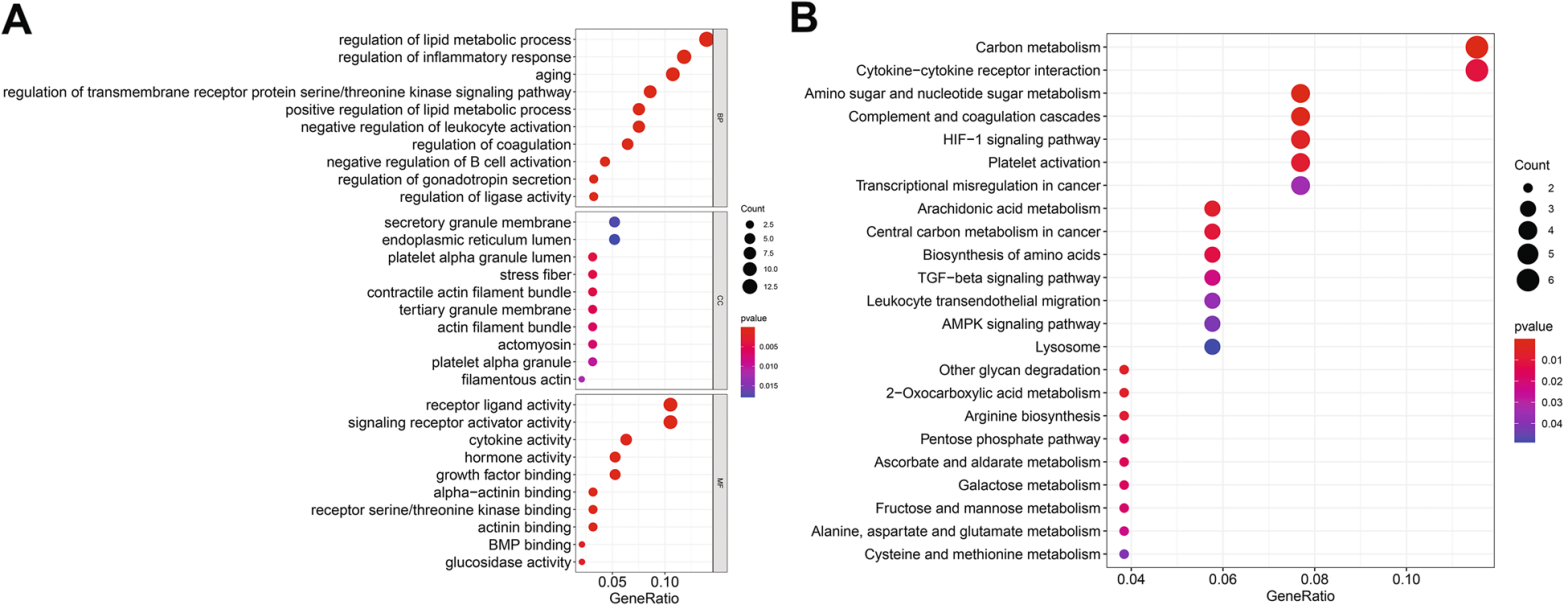
Gene ontology enrichment (**A**) and KEGG (**B**) enrichment analysis were performed in the brown modules

**Fig. 8:**
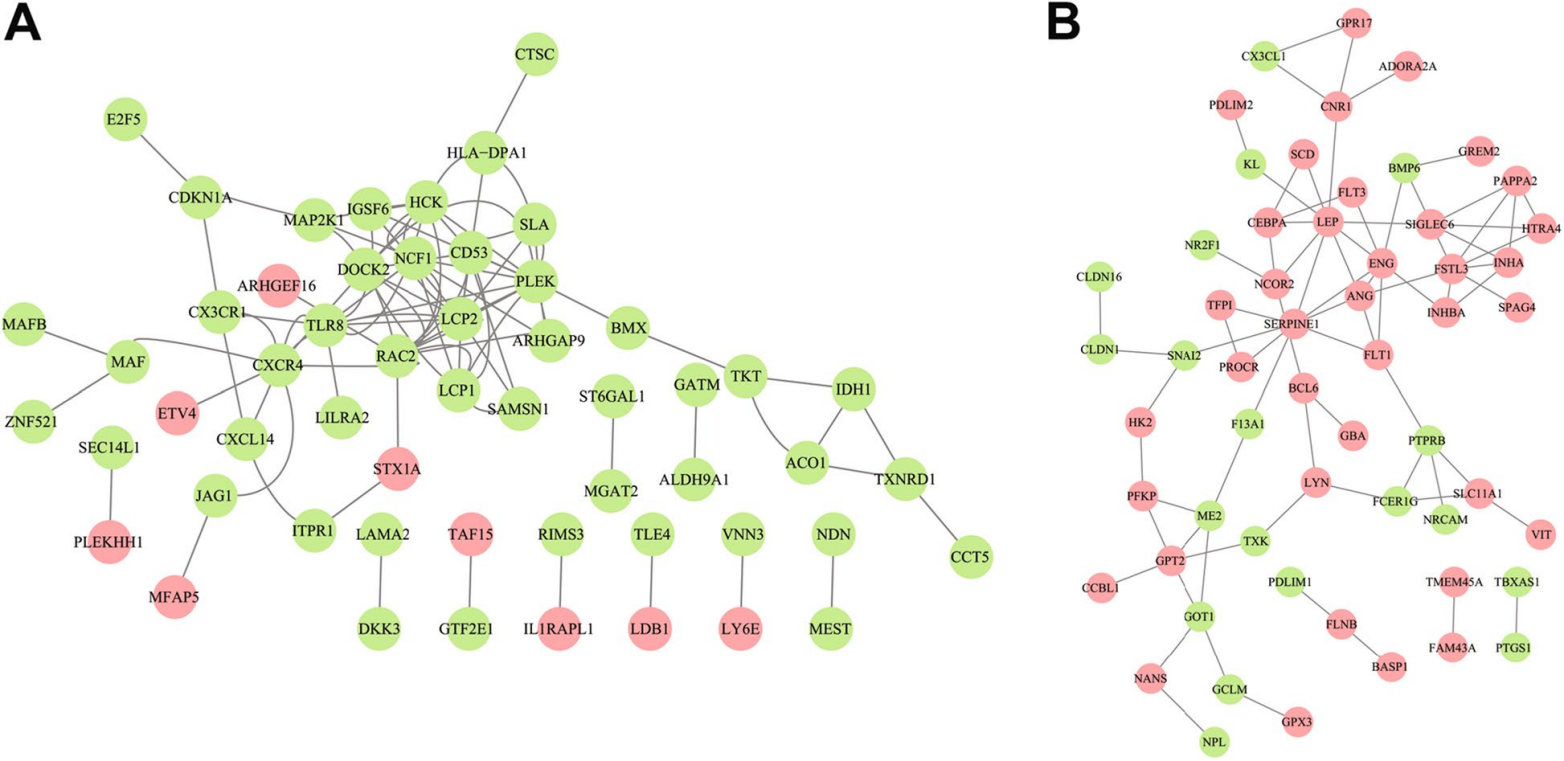
Protein–protein networks of the hub genes in the red (**A**) and brown (**B**) modules

**Fig. 9:**
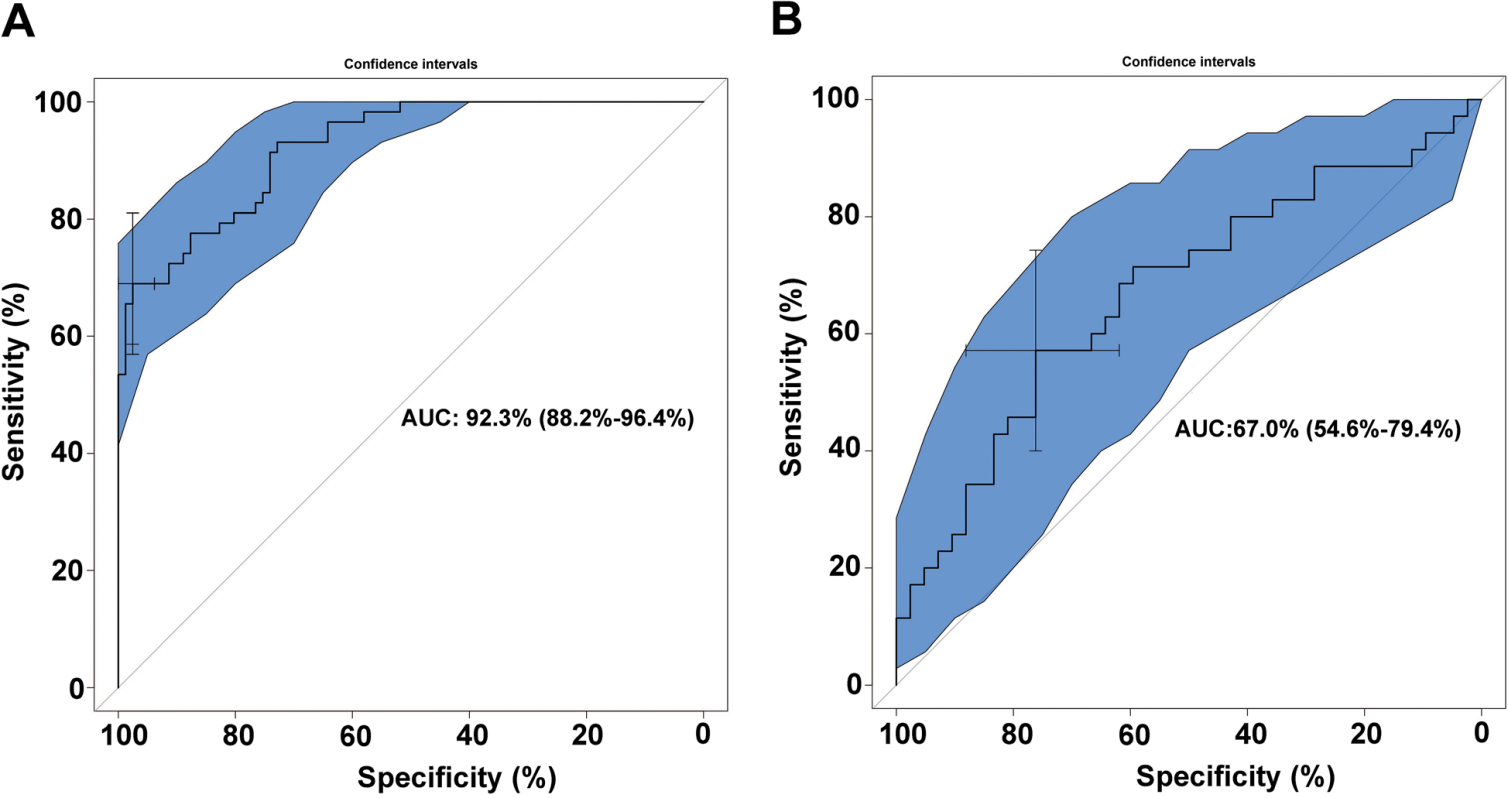
Evaluation of specificity and sensitivity of the hub genes using the receiver operating characteristic in the training dataset (**A**) and external validation dataset (**B**)

**Fig. 10:**
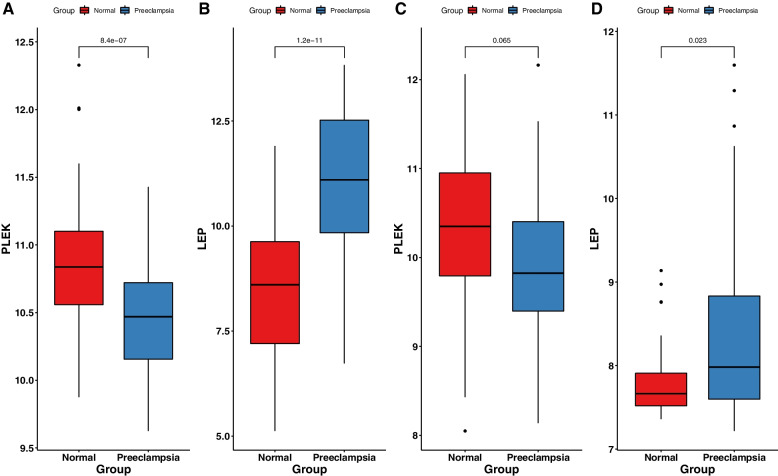
Validation of expression level of the two key genes (PLEK, LEP) in the training dataset (**A**,**B**) and external validation datasets(**C**,**D**)

WGCNA was used to construct gene co-expression networks to discover biologically essential gene modules and better understand genes linked with clinical traits. We selected the genes with the largest variance (25%) to the WGCNA analysis. In this investigation, β = 5 (scale-free R^2^ = 0.90) was selected as the soft threshold to establish a scale-free network. We then applied average-linkage hierarchical clustering method to cluster these genes and six module were obtained, of which yellow and brown modules were the most associated with disease (Fig. 5A-B). We further used MS as the overall gene expression level to assess the correlation with clinical phenotypes. The Fig. 5C and D showed that the correlation between gene significance and clinical trait in yellow and brown modules. According to the GO and KEGG enrichment analyses, the genes in the yellow module were significantly enriched in the NADP metabolic process, cellular aldehyde metabolic process, and other processes (Fig. 6A). Chemokine signalling pathway, Th1 and Th2 cell differentiation, B cell receptor signalling pathway, and oxytocin signalling pathway were all significantly enriched in KEGG enrichment (Fig. 6B). In the brown module, GO enrichment analysis revealed that the genes were significantly enriched in regulation pf lipid metabolic process, aging, cytokine activity, negative regulation of B cell activation, negative regulation of leukocyte activation (Fig. 7A), and cytokine – cytokine receptor interaction, HIF- 1 signalling pathway, TGF—beta signalling pathway, and leukocyte trans-endothelial migration were enriched in KEGG pathway (Fig. 7B). We then constructed a protein–protein interaction network (PPI) from the hub module through the String database and further visualized using the Cytoscape software. Figures 8A and 8B depict the interaction relationship between genes in the yellow and brown modules, respectively. Finally, we used the Maximal Clique Centrality (MCC) algorithm from the cytoscape software to identify 15 hub genes, including LCP1, IGSF6, TLR8, HCK, SLA, DOCK2, RAC2, LCP2, PLEK, CD53, SERPINE1, ENG, ANG, LEP, and FLT1. To restrict the spectrum of core genes, a logistic univariate and multivariate regression analyses were used to construct models associated with PE. Finally, two important genes, PLEK and LEP, were retained with *P* < 0.05 (Table 1). According to ROC analysis, the area under the curve (AUC) value of this model on the merging dataset and external validation dataset achieved 0.923 (Fig. 9A) and 0.670 (Fig. 9B), respectively. PLEK was shown to be significantly expressed in the pooled dataset, although in normal tissues rather than PE tissues (Fig. 10A). However, PLEK was not significantly expressed in the external validation set (*P* = 0.065), which could be attributed to the limited PE sample size in the external dataset (Fig. 10C). Interestingly, the LEP gene was significantly expressed in both the merged dataset and the external validation set, but its expression in normal tissues was lower than in PE tissues (Figs. 10B and D).

**Table 1:** Univariate and multivariate logistic regression

NA	Univariate analysis	NA	Multivariate analysis	NA
	OR (95% CI)	*P* value	*P* value	*P* value
LCP1	0.145 (0.059–0.355)	< 0.001	NA	
IGSF6	0.365 (0.205–0.650)	< 0.001	NA	
TLR8	0.303 (0.141–0.649)	0.002	NA	
HCK	0.257 (0.116–0.567)	< 0.001	NA	
SLA	0.325 (0.158–0.666)	0.002	NA	
DOCK2	0.340 (0.165–0.697)	0.003	NA	
RAC2	0.324 (0.155–0.680)	0.003	NA	
LCP2	0.335 (0.163–0.688)	0.003	NA	
PLEK	0.104 (0.039–0.278)	< 0.001	4.100000e-02 (3.000000e-03–5.150000e-01)	0.013
CD53	0.219 (0.102–0.471)	< 0.001	NA	
SERPINE1	2.040 (1.253–3.321)	0.004	NA	
ENG	4.949 (2.678–9.146)	< 0.001	NA	
ANG	3.438 (1.766–6.691)	< 0.001	NA	
LEP	2.228 (1.698–2.924)	< 0.001	2.524000e + 00 (1.568000e + 00–4.065000e + 00)	0.000
FLT1	2.083 (1.427–3.040)	< 0.001	NA	

Note:

### Immune infiltration analysis

**Fig. 11:**
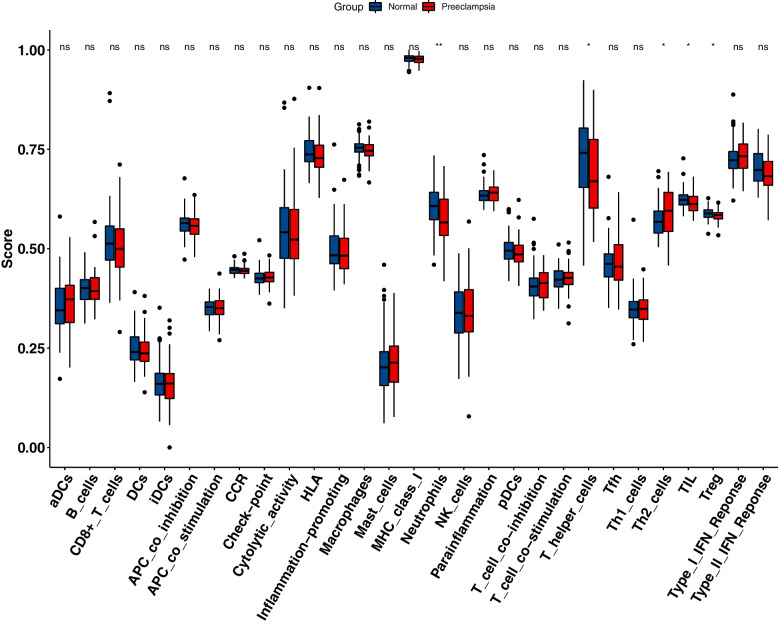
A comparison of the 29 immune cells and functions between normal and PE samples

**Fig. 12:**
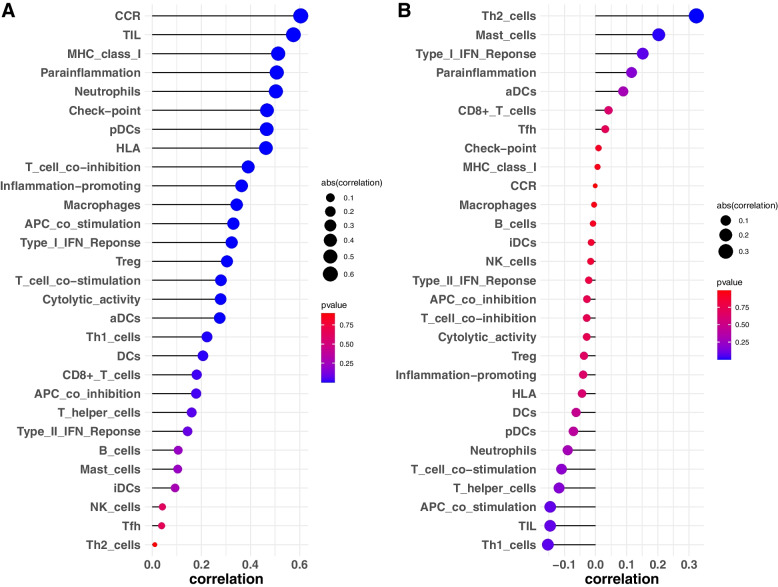
Correlation of the two key genes, including PLEK (**A**) and LEP (**B**), with the 29 immune cells and functions

The immune enrichment value of each sample was calculated using the ssGSEA algorithm, and the wilcoxon-rank test was used to evaluated the immune cell infiltration level between normal samples and PE samples (Fig. 11). In addition, the relationship between PLEK, LEP, and immune infiltration was investigated. We discovered a substantial link between PLEK CCR, TIL, and other immune functions, implying that PLEK may play an important role in immunity, whereas LEP was significantly positively correlated with Th2 cells and negatively correlated with Th1 cells. This suggested that LEP was closely linked to T cell function (Figs. 12A and B).

### RNA extraction and quantitative real-time PCR

**Fig. 13:**
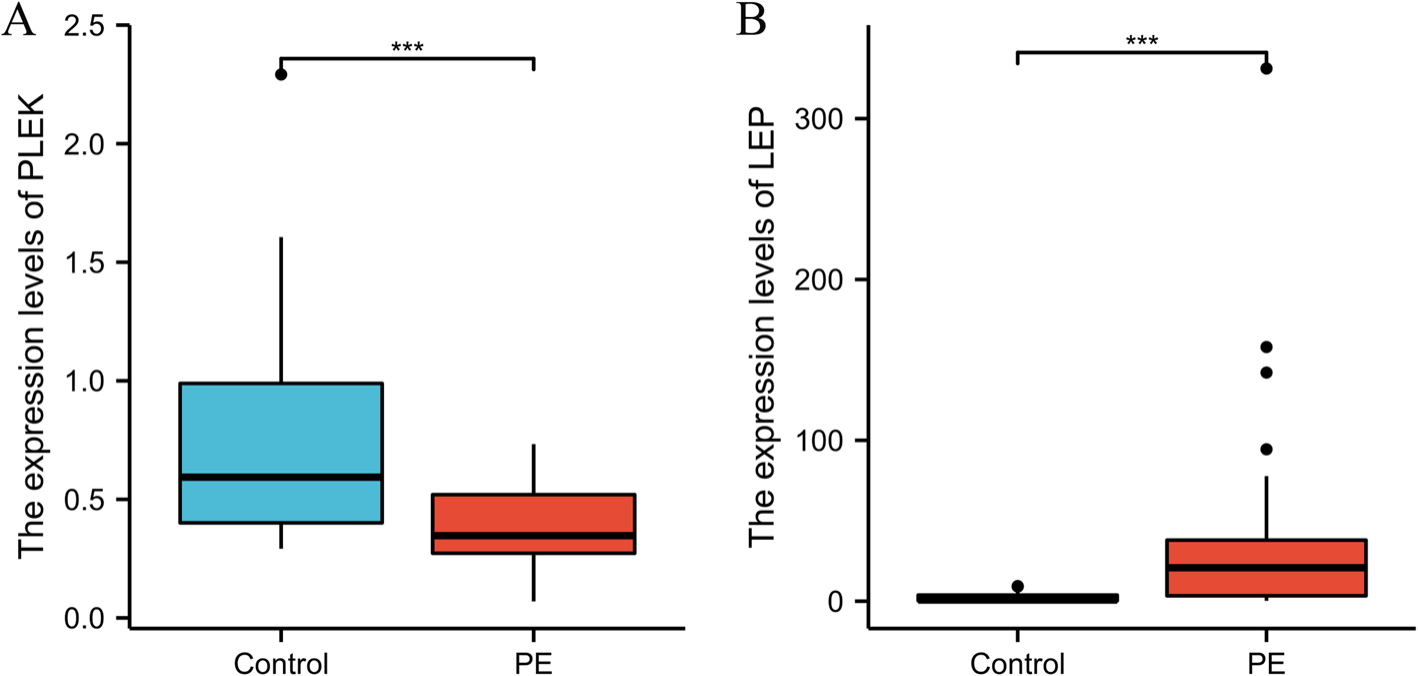
Two key genes show significantly different expression levels in placenta samples from patients with PE and normal healthy controls through the PCR validation. * *P* < 0.05; **P < 0.01

As illustrated in Fig. 13, the PLEK expression levels were significantly lower in the PE samples as compared to normal tissues, while LEP expression levels were significantly higher in normal samples, which was consistent with our analysis results (Figs. 13A and B).

## Discussion

This study employed a multi-step integrated bioinformatics analysis of the chip data to identify the hub gene as a biomarker for PE. 376 down-regulated and 305 up-regulated DEGs were firstly identified using the gene expression profiles of the GSE10588, GSE25906 and GSE48424 datasets. According to these DEGs, we further evaluated prospective drugs for PE treatment through the CMAP database. The analysis of the action mode of 51 compounds revealed that the interaction mechanism for prednisone, dexamethasone, rimexolone, and piretanide occurred via the glucocorticoid receptor agonist. Trazodone, buflomedil, and scoulerine interacted via the adrenergic receptor antagonist, whereas the co-action mechanism of irinotecan, camptothecin, and doxorubicin occurred via topoisomerase inhibitor. Our study identified drugs for PE and may suggest therapeutic targets for future research. However, there is a need to deeply understand whether these treatments are effective in treating PE to provide safe outcomes for the both mother and the foetus.

With the development of the high-through sequencing, a large number of disease-related biomarkers have been identified. Non-coding RNAs play a critical role in the development and biological process of disease [41–43]. Chen et al. reviewed and developed a numerous of computational models to predict the potential non coding RNAs–disease associations. For example, a NCMCMDA and DBNMDA model which applied to predict the potential miRNA–disease associations, showed a high performance than many previous computational methods [44, 45].Cumulative evidence revealed that the unbalanced expression of specific ncRNA is involved in the pathogenesis of PE [46].

However, there is no researches about incorporate the existing predictive model to predict the potential association between non coding RNA and PE presently. Therefore, it is urgent need to applied these model to predict the association between non coding RNA and PE. In our study, trough WGCNA and logistic analysis, we identified two key genes (PLEK and LEP) and constructed a diagnostic model. Leptin (LEP), also known as a placental hormone, is a multifunctional 16 kDa peptide hormone encoded by the LEP gene on chromosome 7 (7q31). It is abundant in adipocytes, the placenta, and tissues such as muscles, liver, brain, and ovaries. LEP is a well-known potential serum marker for PE, particularly in early-onset PE placental tissues [47, 48]. Furthermore, leptin can boost blood supply to the placenta by promoting the formation of new blood vessels [49]. Researchers discovered that leptin can reduce trophoblast cell apoptosis in PE [48], as well as induce endothelial cell proliferation [50], promote immune cell activation, proliferation, and maturation, and prevent monocyte apoptosis. In general, leptin promotes Th-1 response and mediates the production of pro-inflammatory cytokines such as TNF-α, interleukin-2, and interleukin-6. According to Muy-Rivera et al. [51], pregnant women with elevated plasma leptin levels have a 3.8-fold greater risk of developing PE. Leptin has also been shown to raise blood pressure in non-pregnant rabbits [52]. Leptin injection raised ICAM-1 and Eseltin circulation concentrations, resulting in hypertension and proteinuria in pregnant rats [53]. Our findings show that the expression level of this gene was higher in the placenta of the PE group than in the control group, which is consistent with earlier findings. PLEK, another hub gene, was found to be significantly expressed in the merged dataset. Its expression was higher in normal tissues than in PE tissues, although it was not significantly higher in the external validation set. Pleckstrin (PLEK), which is found on human chromosome 2, is a protein kinase C target [54] that is involved in signal transduction and hematopoietic cell differentiation [55]. PLEK play an important role in immune and inflammatory responses [56, 57]. Studies have been shown that PLEK is significantly over-expressed in periodontitis, cardiovascular disease, rheumatoid arthritis, and ulcerative colitis [58], and thought to be a crucial mediator in the secretion and activation pathways of pro-inflammatory cytokines TNF-α and IL-1β [59, 60]. While studies have suggested that LILRA2, EVI2A, and PLEK play a role in recurrent miscarriage [61], the underlying mechanism in placental function and regulation has yet to be fully investigated. This is the first study to examine PLEK expression in PE. An examination of its mechanisms would necessitate additional research.

PE is a complex systemic condition, and it has been shown that the immune system plays a significant role in its development [62]. As a result, we evaluated the landscape of 29 immune cell infiltration levels in PE and control samples. The results indicated that the infiltration level of neutrophils, T helper cells, Th2 cells, TIL, and Treg cells were significantly differed between PE and normal tissues. Immune cell infiltration is a new bioinformatics technique that has been used to investigate the diagnosis and prognosis of kidney cancer [63], malignant glioma [64], breast cancer [65], oral squamous cell carcinoma [66], ulcerative colitis[67], osteosarcoma [68], and variety of other diseases. Nonetheless, it has received little attention in the field of PE. The complicated connection between the maternal immune system and its semi-allogeneic foetus is critical in normal pregnancy. Moreover, establishing and maintaining the maternal and foetal immune balance is a prerequisite for normal pregnancy [69]. Abnormal maternal immunological and inflammatory responses to foetal antigens result in increased release of various toxic cytokines, which causes trophoblast cell invasion, vascular remodelling, and placental implantation disorders. Changes in the innate immune system primarily regulate this inflammatory response, with the adaptive immune system possibly playing a supporting role [70].

T cells are thought to be the most important cells in regulating immunological homeostasis [71, 72]. T lymphocytes account for 1%-3% of decidual immune cells [73]. In a normal pregnancy, the mother exhibits Th2 cell-type immunological tolerance, preventing embryo rejection [74]. However, in PE patients, the Th1/Th2 ratio increases. As a result, the Th1/Th2 balance changes towards Th1 [75]. Aside from the Th1/Th2 imbalance that contributes to the onset and progression of the disease, there is also an imbalance of Th17/ regulatory T cells. This imbalance, which was exacerbated by the Th17 immune bias, also contributed to the development of PE. Th17/ Treg cells are balanced at the maternal-foetal interface during normal pregnancy to preserve maternal immunological tolerance and inhibit the inflammatory response [76]. In our study, we identified most of T cells showed a significant difference between normal and PE samples, reveled that the T cells play an important role in PE.

## Conclusion

PLEK and LEP were identified as two genes implicated in the development and progression of PE in this investigation. Although more in vivo and in vitro validations are needed, our findings help to understand the pathological process of PE and may serve as a theoretical foundation for future research. The functional annotation and pathway enrichment analysis results show that the immunological mechanism is important in the etiology of PE. Also, because of the maternal and infant complications of PE, it is critical to uncover the aetiology and molecular mechanism, develop molecular biomarkers and investigate effective drugs for the early detection, prevention, and personalized treatment of PE.

### Limitations

This study not only points out several benefits of bioinformatics analysis but also highlights some limitations. The dependability of the original microarray dataset is critical to the validity of our conclusions, although the results are constrained due to the small sample size. Similarly, validation results are limited. Second, despite the identification of two hub genes as prospective biomarkers for PE immunotyping, no in vivo and in vitro studies have been conducted. More research on the functions and regulatory mechanisms of key genes in PE is still needed. As a result, this will be the focus of future efforts.

## Supplementary Information

Additional file 1:

## Data Availability

The datasets that support the findings of this study are openly available in the Gene Expression Omnibus (GEO) database under accession ID GSE10588, GSE25906, GSE48424 and GSE60438, [http://www.ncbi.nlm.nih.gov/geo/].

## Abbreviations

PE: Preeclampsia
GEO: Gene Expression Omnibus
DEGs: Differentially expressed genes
WGCNA: Weighted gene co-expression network analysis
PPI: Protein–protein interaction network
CMAP: Connectivity map
TOM: Topological overlap matrix
GS: Gene significance
MEs: Module eigengenes
MM: Module membership
GO: Gene ontology
KEGG: Kyoto Encyclopedia of Genes and Genomes
ssGSEA: Single-sample Gene Set Enrichment Analysis
MOA: Mechanism of actions
ROC: Receiver operating characteristic
AUC: Area under the curve
CCR: Chemokine receptor
TIL: Tumor infiltrating lymphocyte
MCC: Maximal clique centrality
